# Topical Formulation Containing Naringenin: Efficacy against Ultraviolet B Irradiation-Induced Skin Inflammation and Oxidative Stress in Mice

**DOI:** 10.1371/journal.pone.0146296

**Published:** 2016-01-07

**Authors:** Renata M. Martinez, Felipe A. Pinho-Ribeiro, Vinicius S. Steffen, Thais C. C. Silva, Carla V. Caviglione, Carolina Bottura, Maria J. V. Fonseca, Fabiana T. M. C. Vicentini, Josiane A. Vignoli, Marcela M. Baracat, Sandra R. Georgetti, Waldiceu A. Verri, Rubia Casagrande

**Affiliations:** 1 Departamento de Ciências Farmacêuticas, Universidade Estadual de Londrina-UEL, Avenida Robert Koch, 60, Hospital Universitário, 86039–440 Londrina, Paraná, Brasil; 2 Departamento de Ciências Patológicas, Universidade Estadual de Londrina-UEL, Rodovia Celso Garcia Cid, Km 380, PR445, Cx. Postal 10.011, 86057–970 Londrina, Paraná, Brasil; 3 Departamento de Ciências Farmacêuticas, Faculdade de Ciências Farmacêuticas de Ribeirão Preto-USP, Av. do Café s/n, 14049–903 Ribeirão Preto, São Paulo, Brasil; 4 Farmacore Biotecnologia LTDA, Rua Edson Souto, 738—Anexo I, Lagoinha, 14095–250 Ribeirão Preto, São Paulo, Brasil; 5 Departamento de Bioquímica e Biotecnologia, Centro de Ciências Exatas, Universidade Estadual de Londrina, Rodovia Celso Garcia Cid, Km 380, PR445, Cx. Postal 10.011, 86057–970 Londrina, Paraná, Brazil; University of Alabama at Birmingham, UNITED STATES

## Abstract

Naringenin (NGN) exhibits anti-inflammatory and antioxidant activities, but it remains undetermined its topical actions against ultraviolet B (UVB)-induced inflammation and oxidative stress *in vivo*. The purpose of this study was to evaluate the physicochemical and functional antioxidant stability of NGN containing formulations, and the effects of selected NGN containing formulation on UVB irradiation-induced skin inflammation and oxidative damage in hairless mice. NGN presented ferric reducing power, ability to scavenge 2,2′-azinobis (3-ethylbenzothiazoline- 6-sulfonic acid) (ABTS) and hydroxyl radical, and inhibited iron-independent and dependent lipid peroxidation. Among the three formulations containing NGN, only the F3 kept its physicochemical and functional stability over 180 days. Topical application of F3 in mice protected from UVB-induced skin damage by inhibiting edema and cytokine production (TNF-α, IL-1β, IL-6, and IL-10). Furthermore, F3 inhibited superoxide anion and lipid hydroperoxides production and maintained ferric reducing and ABTS scavenging abilities, catalase activity, and reduced glutathione levels. In addition, F3 maintained mRNA expression of cellular antioxidants glutathione peroxidase 1, glutathione reductase and transcription factor Nrf2 (nuclear factor erythroid 2-related factor 2), and induced mRNA expression of heme oxygenase-1. In conclusion, a formulation containing NGN may be a promising approach to protecting the skin from the deleterious effects of UVB irradiation.

## Introduction

The skin is a physical barrier between the organism and the environment being constantly challenged by deleterious effects of UV solar radiation, mainly ultraviolet B (UVB) irradiation [1]. There are systemic and local effects of skin UVB irradiation [2]. For instance, UVB irradiation of the skin induces systemic effects such as activation of the hypothalamic–pituitary–adrenal axis [3]. Locally, skin cells produce a great variety of regulatory molecules interacting with immune cells and are also affected by neurohormonal axis [2,3]. UVB irradiation triggers the overproduction of reactive oxygen species (ROS) and depletion of endogenous antioxidants such as reduced glutathione (GSH), glutathione peroxidase (Gpx), and catalase in the skin. As a result, there is skin inflammation [4,5]. Importantly, accumulated damage resulting from chronic UVB irradiation exposure has been shown to cause skin cancer and premature skin aging [4,6].

UVB induces overproduction of ROS such as superoxide anion (O_2_^•-^) and singlet O_2_ (^1^O_2_), which are critical events for the onset of oxidative stress conditions [7–9]. *In vitro*, even three min after UVB irradiation there is detectable production of O_2_^•-^ and ^1^O_2_ peaking after 15 and 30 min of UVB irradiation [9]. The mitochondria electron-transport chain Complex I and Complex III produce O_2_^•-^ and ^1^O_2_ [10,11]. On the other hand, there is also evidence that UVB irradiation induces O_2_^•-^ production via phagocytic NADPH oxidase 2 (NOX2) complex [7,8], cyclooxygenase and lipoxygenase [12,13] independently of the mitochondria electron-transport chain [14]. Moreover, the NOX2- and cyclooxygenase-dependent production of O_2_^•-^ induced detectable NFκB translocation to the cell nucleus (NFκB activation) within 15 min [14]. Therefore, the mitochondria electron-transport chain-dependent and NOX2/cyclooxygenase-dependent production of O_2_^•-^ occurs within min after UVB irradiation [9,14] indicating that both mechanisms are important to O_2_^•-^ production and their relative roles might depend on experimental conditions.

O_2_^•-^ is an important source of additional ROS. For instance, O_2_^•-^ reacts with hydrogen peroxide (H_2_O_2_) generating the cytotoxic hydroxyl radical (^•^OH) [15]. In turn, ^•^OH causes lipid peroxidation (LPO) process, a well-established detrimental consequence of UVB exposure that induces pro-inflammatory products [16]. Thus, ROS contribute to pro-inflammatory signaling cascades and consequent production of cytokines such as interleukin-1β (IL-1β), and tumor necrosis factor-alpha (TNF-α) [17–20]. Importantly, there is reciprocal activation by oxidative stress and inflammatory mediators because cytokines such as TNF-α also induce O_2_^•-^ production [21], activate or induce the expression of inflammatory enzymes such as NOX2 and cyclooxygenase [7,8,12,13] and trigger inflammation in a NADPH oxidase-dependent manner [18]. Therefore, ROS and cytokines act in synergism during inflammatory skin diseases [22].

Epidemiological studies have indicated that sunscreens do not fully prevent UVB irradiation-induced skin injuries. Thus, novel chemopreventive treatments need to be identified [23]. Considering the synergic effect of ROS and inflammatory mediators, the use of molecules with antioxidants and anti-inflammatory effects is a promising approach to inhibit UVB irradiation-induced skin damage [24–29]. In this context, much attention has been paid to antioxidants from natural sources [24,25,30]. Moreover, naturally occurring agents are considered to be less toxic, of low cost, and more effective in controlling various human malignancies [31,32].

Flavonoids represent the most common and widely distributed group of plant phenolics [33]. These molecules have a structural inherent antioxidant activity and anti-inflammatory properties [33], which strongly suggest the potential of these compounds to inhibit UVB irradiation-induced skin damaging events [31]. Therefore, their topical use may provide the necessary photochemical protection in addition to human sunscreens. In fact, topical delivery of active biomolecules is considered a promising strategy to reduce UVB irradiation-induced skin damages [34]. Moreover, the topical use of bioactive substances is a powerful strategy to avoid possible systemic toxicity.

Molecules of the flavonoid subgroup flavanones are found almost exclusively in citrus fruits and comprise the most common plant polyphenolic compounds in the human diet [35]. Naringenin (NGN) (5,7,4’-trihydroxyflavanone) is one of the most abundant flavanones found in fruits such as grapefruit, lemon, tangerine and orange [36]. It has many pharmacological activities such as anti-atherogenic, anti-cancer, antioxidant, and anti-inflammatory [36–43]. NGN protects HaCaT human keratinocytes against UVB-induced carcinogenesis and aging [37]. Importantly, systemic administration of NGN inhibits UVB-induced skin oxidative stress and inflammation *in vivo* [44]. Recently, two topical formulations containing NGN were developed; NGN-loaded elastic liposome [45] and submicron-carrier NGN formulation [46]. These NGN formulations did not induce skin-irritating effects [45,46]. However, there is no evidence on *in vivo* topically active formulation containing NGN to preventing photodamage. The present study aimed to: 1) select a suitable *in vitro* antioxidant activity test for functional stabilities studies of NGN topical formulations; 2) prepare and evaluate the physicochemical and functional stabilities of topical formulations containing NGN; and 3) investigate the *in vivo* protection of the most stable formulation containing NGN against UVB-induced skin inflammation and oxidative stress.

## Material and Methods

### Chemicals

2,2′-azinobis (3-ethylbenzothiazoline- 6-sulfonic acid) (ABTS), reduced glutathione (GSH), 5,5’dithio-bis-(2-nitrobenzoic acid) and nitroblue tetrazolium (NBT) were obtained from Sigma Chemical Co. (St. Louis, MO, USA). Naringenin at 95% from Santa Cruz Biotechnology (Dallas, TX, USA). Tert-butyl hydroperoxide and 2-deoxy-D-ribose from Acros (Pittsburgh, PA, USA). Enzyme-linked immunosorbent assay (ELISA) kits from eBioscience (San Diego, CA, USA). Superscript® III, Oligo(dT)12-18 primers, Platinum SYBRGreen® and primers from Invitrogen (Carlsbad, CA, USA). Materials for formulations were from Galena (Campinas, SP, Brazil). All other reagents used were of pharmaceutical grade.

### *In vitro* antioxidant activity of NGN

#### FRAP assay

The FRAP assay was used to determine the ferric reducing antioxidant power of NGN (60 μg/mL) at 595 nm [47]. A standard curve with trolox (4.0–20.0 μmol/L) allowed calculating the results in μmol/L of trolox equivalent per μg/mL of sample.

#### ABTS assay

The ABTS scavenging ability of NGN (0.125–2 μg/mL) was determined by the decrease in absorbance at 730 nm [48]. The following equation was applied: Equation I: % of activity = (1- sample absorbance/control absorbance) x 100.

#### Hydroxyl assay

The ^•^OH scavenging ability was measured by the reduction of thiobarbituric acid reactive substances (TBARS) formed upon the degradation of deoxyribose by ^•^OH generated in Fenton reaction [47–49]. The scavenging of hydroxyl free radical by NGN (25–500 μg/mL) was calculated by equation I.

#### Iron-independent lipid peroxidation

The inhibitory activity of lipid peroxidation of NGN (10–500 μg/mL) was determined by decreasing the production of lipid hydroperoxides, a primary product of lipid peroxidation [48]. The following equation was used:

% Activity = 1- (absA after incubation—absA without incubation) / (absC after incubation—absC without incubation) x 100. AbsA is the absorbance of a sample, and absC is the absorbance of the control.

#### Iron-dependent lipid peroxidation

Mitochondria of hairless mice were used as a source of lipid membranes to evaluate lipid peroxidation and were prepared by standard differential centrifugation techniques. The ability of graded concentrations of NGN (2.5–500 μg/mL) to inhibit iron-induced lipid peroxidation was evaluated by reduction of TBARS formation [48,50]. The inhibition of iron-dependent lipoperoxidation was calculated by equation I.

### Formulations

Formulations F1, F2, and F3, were prepared to vary the content of excipients (Table 1). Self-emulsifying agents were Polawax^®^, Hostacerin SAF^®^ or Net FS^®^. Carbopol^®^ 940 was used as stabilizing agent. Caprylic/capric triglyceride was used as the emollient and propylene glycol as solubilizing agent and moisturizer. Phenonip was used as the preservative and deionized water was used for the preparation of all formulation. NGN (0.5%) was solubilized in propylene glycol and then added to the formulations at room temperature. Control formulations did not contain NGN.

**Table 1:** Percent composition (weight/weight) of formulation F1, F2, and F3.

Components	F1	F2	F3
**Polawax**^**®**^^**a**^	10	_	_
**Hostacerin SAF**^**®**^^**b**^	_	5	_
**Net FS**^**®**^^**c**^	_	_	2
**Caprylic/capric triglyceride**	5	5	5
**Carbopol**^**®**^ **940 (1%)**^**d**^ (qsp)*	_	_	100
**Triethanolamine**	_	_	0.5
**Propylene glycol**	6	6	6
**Phenonip**	0.4	0.4	0.4
Deionized water (qsp)*	100	100	_

*Quantity sufficient for preparation

^a^ Self-emulsifying wax (Cetostearyl alcohol and polyoxyethylene derived from a fatty acid ester of sorbitan 20 0E);

^b^ Self-emulsifying was prepared without heating (Ammonium acryloyldimethyl-taurate/VP copolymer and rapeseed oil sorbitol esters and trilaureth-4 phosphate and mineral oil and isopropyl palmitate);

^c^ Self-emulsifying was prepared without heating (Polyglyceryl-10 Myristate and Triethylhexanoin and Glycerin and Water);

^d^ Carboxypolymethylene.

### Physicochemical and functional stability of formulations

Regarding stability studies, formulations were packaged in semipermeable polypropylene containers and stored at 4°C and 40±2°C/ 75±5% of relative humidity (RH) for 6 months. Samples were evaluated 0, 30, 60, 90, and 180 days after preparation [51]. NGN raw material was also stored in the same storage conditions to evaluate its functional stability. The physicochemical and functional stability were determined at room temperature by the following methods.

#### Organoleptic test

The examination of organoleptic features of the samples were at the same temperature, lighting, and packaging conditions to assess variations in appearance, phase separation, color and smell [52].

#### pH measurements

One gram of each formulation was weighted and diluted with deionized water to a volume of 10 mL. After homogenization, the pH of the samples was measured using a digital pH meter [53].

#### Centrifugation assay

Samples weighing 2 g were taken and centrifuged at 1660× *g* for 30 min. After centrifugation of samples, the separation of the dispersed phase due to either creaming or coalescence was observed [53].

#### Functional stability

The functional stability [51] was measured by ABTS method as described in section “2.2.2. ABTS assay”. Formulations containing NGN were diluted in ethanol to obtain the concentration of 0.8 μg/mL. It was the sample concentration used for the analysis of NGN raw material in the reaction medium. A positive control in the absence of sample and a positive control added with formulations without NGN were used.

After the stability studies, the *in vivo* efficacy of the most stable formulation containing NGN against skin inflammation and oxidative stress caused by UVB irradiation was evaluated.

### *In vivo* efficacy of topical formulation containing NGN

#### Animals

*In vivo* experiments were performed in sex matched hairless mice (HRS/J), weighing 20–30 g, obtained from the University Hospital of Londrina State University. Mice had free access to water and food at a temperature of 23°C ± 2 and a 12 h light and 12 h dark cycles. The Animal Ethics Committee (CEUA process number 19972.2013.46) of the Londrina State University approved all procedures of this study.

#### Experimental protocol

Hairless mice were randomly designed to groups with 5 mice each as follows: non-irradiated control, irradiated control, irradiated and treated with formulation without NGN, irradiated and treated with the formulation containing NGN (F3). Mice received topical treatment on the dorsal surface with 0.5 g of the formulation, 12 h, 6 h, and 5 min before, and 6 h after the beginning of irradiation session.

#### Irradiation

The UVB source used was a Philips TL/12 RS 40W (Medical-Holand) emitting a continuous spectrum between 270 and 400 nm with a peak emission at 313 nm [24,47]. There was 20 cm between the lamp and mice position with an irradiation of 0.384 mW/cm^2^. An IL 1700 radiometer (Newburyport, MA, USA) equipped with the sensor for UV (SED005) and UVB (SED240) was used to determine the irradiation intensity. The irradiation dose used to induce skin inflammation was 4.14 J/cm^2^ [24,47]. Groups were irradiated simultaneously. Mice were terminally anesthetized with 1.5% isoflurane (Abbott [Abbott Park, IL, USA]) 12 h (for edema, GSH, FRAP and ABTS tests), 2 h (for catalase and NBT tests) or 4 h (for cytokine, lipid peroxidation and qPCR tests) after the UVB exposure. Afterward, the full thickness of the dorsal skins was removed. In the tests of 2 h and 4h after the UVB exposure, mice were decapitated immediately after anesthetization, and dorsal skin samples were collected. Samples were stored at -70°C until analysis. Samples collected for cutaneous edema determination were weighed immediately after removal and were not frozen.

#### Skin edema

The skin edema was measured as an increase in the dorsal skin weight. After dorsal skin removal, a constant area was delimitated with the aid of a mold, followed by weighing [24,47]. The result is expressed in mg of skin.

#### Cytokine measurement

Skin samples were homogenized in 500 μL of saline solution using Tissue-Tearor (Biospec). The homogenates were centrifuged (2,000 *g*, 15 min, 4°C) and supernatants were stored at -70°C. Supernatants were used to measure the cytokine levels by an enzyme-linked immunosorbent assay (ELISA) according to manufacturer’s instructions (eBioscience) [47]. Absorbance was determined at 450 nm using a microplate spectrophotometer reader (Multiskan GO, Thermo Scientific) and the results are expressed as picograms (pg) of each cytokine/mg of skin.

#### FRAP assay

The reducing ability of skin sample was determined by FRAP assay [51]. The samples of skin were homogenized in 400 μL of KCl (1.15%) using a Tissue-Tearor (Biospec) and centrifuged (1,000 *g*, 10 min, 4°C). The supernatant was employed to measure the antioxidant capacity of skin. Supernatant (30 μL) was mixed with the FRAP reagent (0.3 mM acetate buffer pH 3.6, 10 mM 2,4,6-Tris(2-pyridyl)-*s*-triazine in 40 mM hydrochloride acid, and 20 mM ferric chloride). The absorbance was determined at 595 nm in a microplate reader (EnSpire, Perkin Elmer). The results were compared to a trolox curve (0.01–20 nmol) and presented as nmol trolox equivalent per mg of skin.

#### ABTS assay

The ABTS radical scavenging ability of skin was measured by the decrease in absorbance at 730 nm [51]. Skin of hairless mice was homogenized in 400 μL of KCl (1.15%) using a Tissue-Tearor (Biospec) and centrifuged (1,000 *g*, 10 min, 4°C). The supernatant was employed to measure the antioxidant capacity of skin. The solution of ABTS was prepared with 7 mM of ABTS and 2.45 mM of potassium persulfate diluted with phosphate buffer pH 7.4 to an absorbance of 0.7–0.8 in 730 nm was prepared. The supernatant (7 μL) was mixed with ABTS solution, and the absorbance was determined after 6 min at 730 nm in a microplate reader (EnSpire, Perkin Elmer). The results were compared to a trolox curve (0.01–20 nmol) and the results are presented as nmol trolox equivalent per mg of skin.

#### Catalase assay

The analysis of catalase activity was evaluated by measuring the decay in the concentration of H_2_O_2_ and the generation of oxygen as described previously [54]. Skin of samples were homogenized in 500 μL of 0.02 M EDTA using a Tissue-Tearor (Biospec), and centrifuged twice (2,700 *g*, 10 min, 4°C). The reaction mixture contained 10 μL of sample, 160 μL of buffer Tris-HCl 1 M with EDTA 5 mM (pH 8.0), 20 μL of deionized water and 20 μL of H_2_O_2_ 200 mM. The catalase activity was determined through the difference between the initial reading and the reading conducted 30 seconds after the addition of H_2_O_2_ at 240 nm in a microplate reader (EnSpire, Perkin Elmer) at 25°C. The catalase values were expressed as unit of catalase/mg of skin/minute.

#### Lipid peroxidation (LPO)

LPO was measured by tert-butyl lipid hydroperoxides-initiated chemiluminescence according to an adaptation of the technique described previously [55]. This test lays on the premise that there is an increase in chemiluminescence associated with oxidative stress leading to the consumption of the antioxidant defenses from the formation of hydroperoxides. Samples of skin were homogenized in 800 μL of phosphate buffer (pH 7.4) using a Tissue-Tearor (Biospec), centrifuged (700 *g*, 2 min, 4°C). Then, 250 μL of the supernatant were diluted in 1730 μL reaction medium (120 mM KCl, 30 mM phosphate buffer, pH 7.4) and mixed with 20 μL of 3 mM tert-butyl hydroperoxide. The reading was conducted in a β-counter Beckman®LS 6000SC (Fullerton, CA, USA) in a non-coincident counting for 30 s with a response range between 300 and 620 nm. The vials were kept in the dark up to the moment of the assay, and determinations were obtained in dark to avoid vial phosphorescence activated by light. The experiment was conducted at 30°C for 120 min. The results were measured in counts per min (cpm) per mg of skin.

#### Superoxide anion (O_2_^•-^) production

The measurement of O_2_^•-^ production in the skin was performed using the NBT assay as described previously [24]. Samples of skin were homogenized in 500 μL of 0.02 M EDTA using a Tissue-Tearor (Biospec) and centrifuged (2000 *g*, 20 seconds, 4°C). Then, 50 μL of the supernatant were incubated in 96-well plate for 1 h. The non-adherent/non-precipitated supernatant was carefully removed, followed by addition of 100 μL of NBT (1 mg/ml) to each well and incubated for 15 min. NBT reaction medium was then carefully removed followed by fixation in methanol 100%. Formazan particles were dissolved by adding 120 μL of KOH 2M and 140 μL of dimethylsulfoxide. Reduction of NBT to formazan was measured at 600 nm using a microplate spectrophotometer reader (Asys Expert Plus, Biochrom) and the results are presented as optical density (OD) per 10 mg of skin.

#### GSH assay

GSH was determined as described previously [47]. Samples of skin were homogenized in 0.02 M EDTA using a Tissue-Tearor (Biospec). Whole homogenates were treated with 50% trichloroacetic acid and were centrifuged twice (2,700 *g*, 10 min, 4°C). The reaction mixture contained 50 μL of sample, 100 μL of 0.4 M Tris and 5 μL 5,5’dithio-bis-(2-nitrobenzoic acid) (1.9 mg/mL in methanol). The absorbance was read at 405 nm (Multiskan GO, Thermo Scientific). A standard curve of GSH (5–150 μM) allowed the analysis of data and presentation as μM of GSH per mg of skin.

#### Reverse transcriptase (RT) and quantitative polymerase chain reaction (qPCR)

Skin samples were homogenized in TRIzol® reagent (Life Technologies), and total RNA was isolated according to manufacturer’s directions. RNA purity was confirmed by the 260/280 ratio. RT-PCR and quantitative PCR were performed using GoTaq® 2-Step RT-qPCR System (Promega) on a StepOnePlusTM Real-Time PCR System (Applied Biosystems®). The relative mRNA expression was measured using the comparative 2^- (∆∆Cq)^ method. The expression of GAPDH mRNA expression was used as a control for tissue integrity in all samples. The primers used were: *Gpx1*, sense: CCAACACCCAGTGACGACC, antisense: CTCAAAGTTCCAGGCAATGTC; *Gr*, sense: TGCGTGAATGTTGGATGTGTACCC, antisense: CCGGCATTCTCCAGTTCCTCG; *Nrf2*, sense: TCACACGAGATGAGCTTAGGGCAA, antisense: TACAGTTCTGGGCGGCGACTTTAT; *HO-1*, sense: CCCAAAACTGGCCTGTAAAA, antisense: CGTGGTCAGTCAACATGGAT; and *Gapdh* sense: CATACCAGGAAATGAGCTTG, Antisense: ATGACATCAAGAAGGTGGTG [24].

### Statistical analysis

Results were analyzed by GraphPad Prism® 4 software and expressed as mean ± standard error mean (SEM). *In vitro* data represent triplicate analysis per experiment and are representative of two separate experiments. The concentration of NGN necessary to inhibit the oxidative process by 50% (IC_50_) was determined using hyperbolic curve. *In vivo* results are mean ± SEM of 5 mice per group per experiment and are representative of two separate experiments. The differences were evaluated by ANOVA followed by Tukey’s *t* test. Statistical differences were considered to be significant at p < 0.05.

## Results

### *In vitro* antioxidant activity of NGN

**Fig 1:**
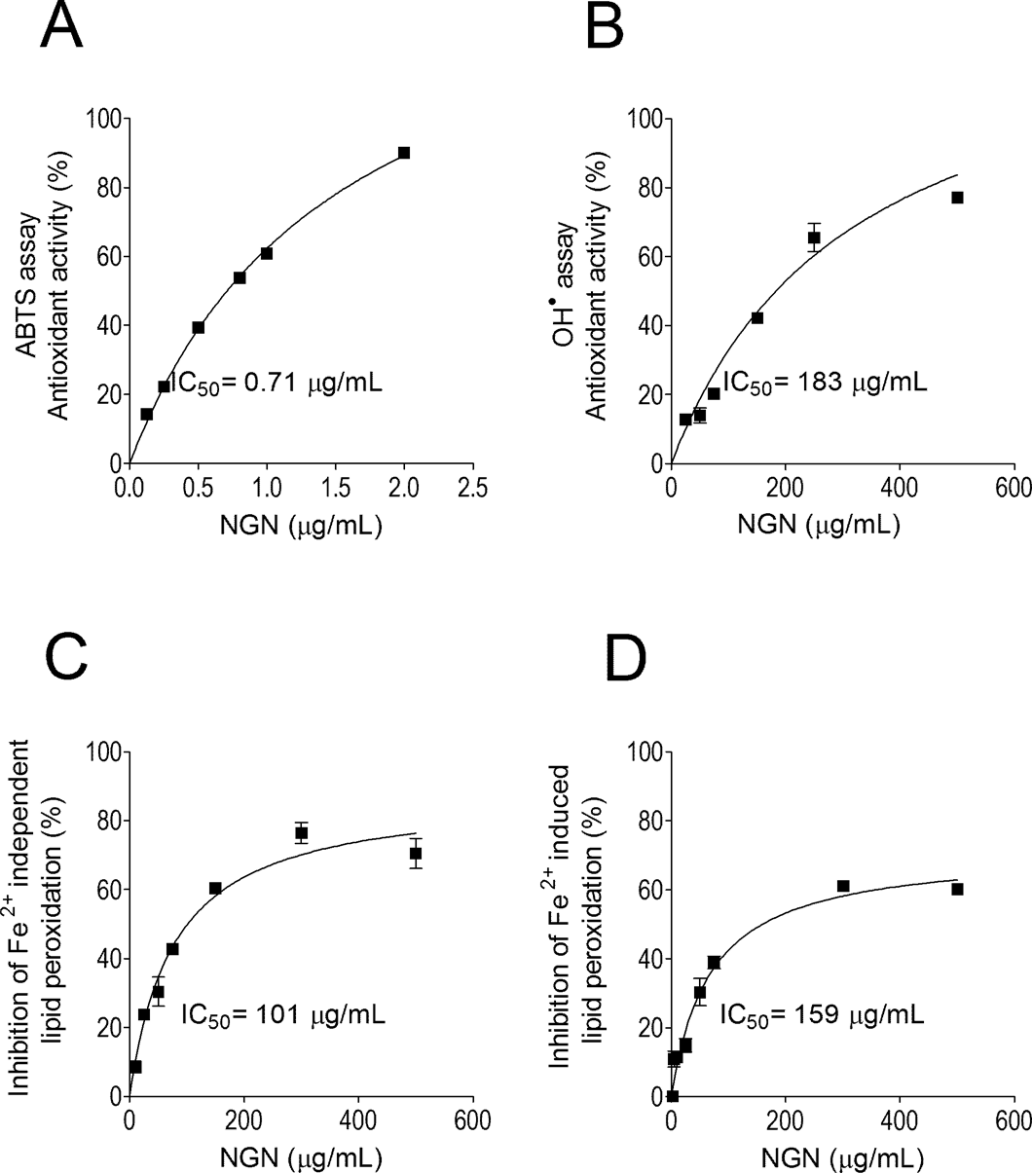
*In vitro* antioxidant activity of NGN. NGN was added at indicated concentration and assayed for scavenging the radical ABTS (Panel A), ^•^OH (Panel B), iron-independent lipid peroxidation (Panel C) and iron-induced lipid peroxidation (Panel D). Results (percentage of inhibition comparing to control) represent means ± SEM of triplicate values representative of two separate experiments.

The antioxidant activity of NGN was evaluated by FRAP, ABTS, ^•^OH, iron-independent and iron-dependent LPO assays. In the FRAP assay, NGN reducing power was 0.157 μmol/L trolox equivalent/μg/mL. NGN concentration-dependently scavenged the ABTS synthetic and ^•^OH radicals with IC_50_ of 0.71 μg/mL and 183 μg/mL, respectively (Fig 1A and 1B). NGN also concentration-dependently inhibited *in vitro* iron-independent and iron-dependent LPO with IC_50_ of 101 μg/mL and 159 μg/mL, respectively (Fig 1C and 1D).

### Preparation and stability of NGN containing formulation

Three topical formulations containing NGN were prepared, and their physicochemical and functional stability under 4°C and 40±2°C / 75±5% RH were evaluated. After six months at 4°C, F1, F2 and F3 (control and added with NGN) maintained their color and consistency characteristics. At 40±2°C/75±5% RH there was no color change in all formulations while there was consistency decrease of F1 and F2 (control and added with NGN). The pH values of F1, F2 and F3 remained compatible with the pH values of skin range from 5.0 to 6.0 [56]. All formulations also remained physically stable upon centrifugation assay, showing no phase separation in the two storage conditions evaluated.

**Fig 2:**
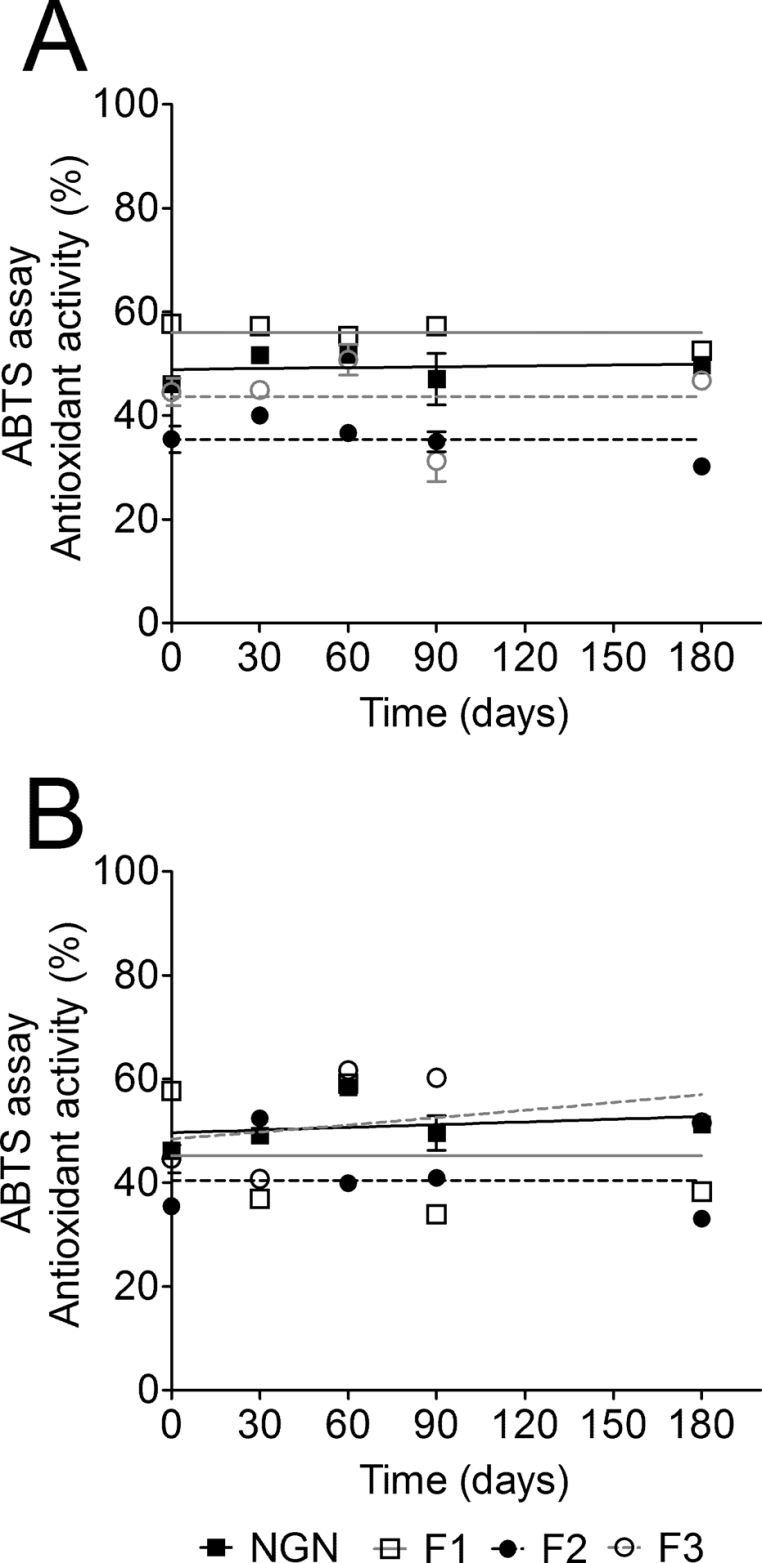
Naringenin (NGN) containing formulations functional stability. F1, F2, and F3 containing NGN storage was at 4°C (A) and 40°C (B) /75% RH for 6 months. The functional stability of NGN containing formulations was determined by the ABTS radical scavenging activity. Results are mean ± SEM.

Regarding functional stability study using ABTS scavenging activity, it was observed that the temperature, storage time and formulation composition influenced the antioxidant activity of NGN. After six months stored at 4°C the ABTS radical scavenging ability of F1 and F2 decreased by 8.94% and 14.88%, respectively. On the other hand, the antioxidant activity of F3 increased 4.98% (Fig 2A). At 40±2°C/75±5% RH, F1 and F2 lost 33.55% and 6.65% of their antioxidant activity, respectively (Fig 2B). On the other hand, the antioxidant activity of F3 increased by 15.77% (Fig 2B). A possible explanation is that F3 is approximately 86% of water and water loss when stored at high temperatures would explain this increase in antioxidant activity. It is important to mention that starting at the beginning of the study, ABTS radical scavenging ability of F2 was lower than NGN raw material. Taking into account all stability results, F3 demonstrated to be the most stable formulation rendering it eligible for evaluation of *in vivo* efficacy of NGN against skin inflammation and oxidative stress caused by UVB irradiation.

### NGN containing formulation reduces UVB irradiation-induced skin edema

**Fig 3:**
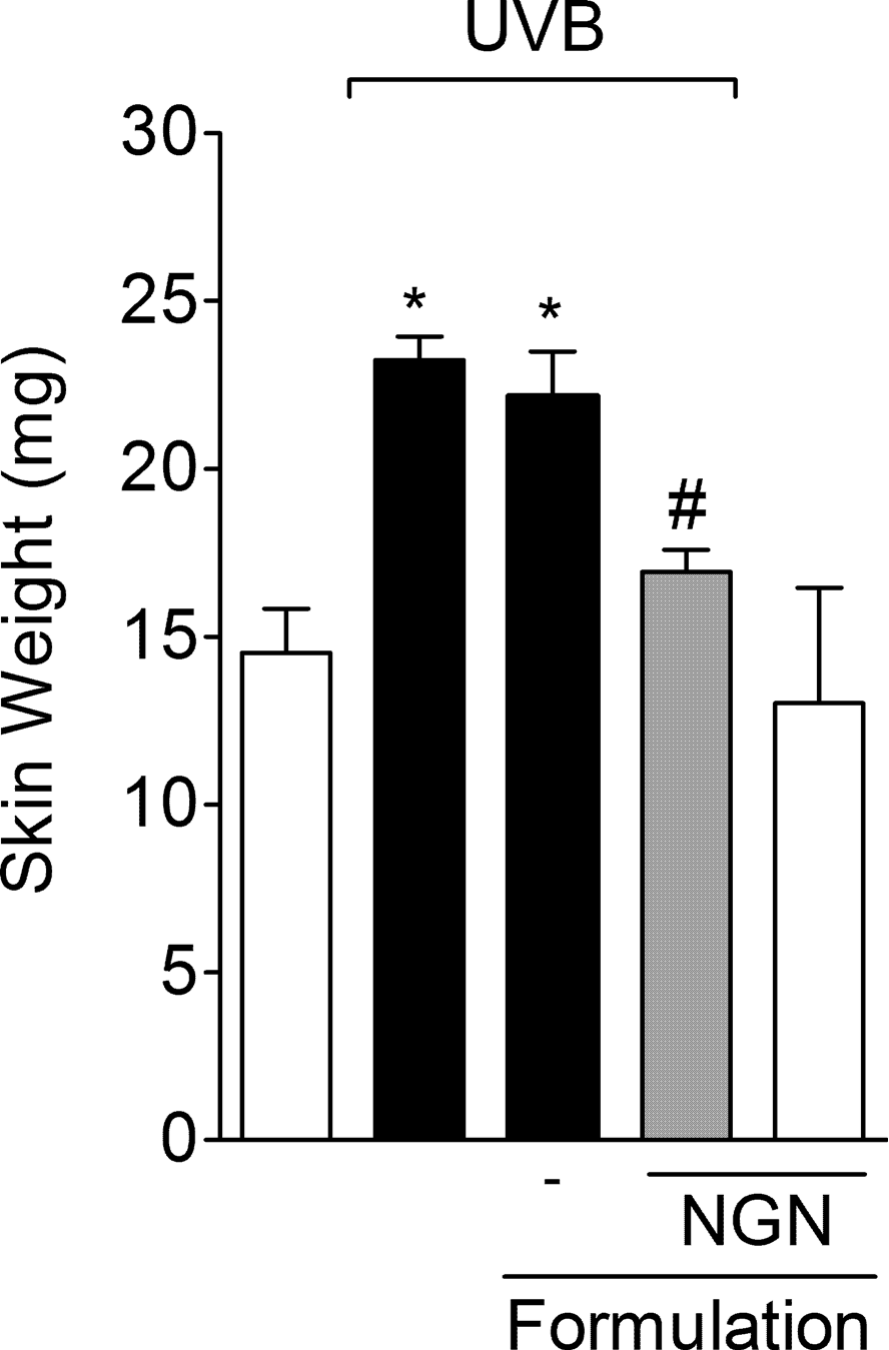
Naringenin (NGN) containing formulation reduces skin edema induced by UVB irradiation. Samples of dorsal skin were collected 12 h after the end of irradiation and used to measure the edema. Bars represent means ± SEM of 5 mice per group per experiment and are representative of two separate experiments. *p<0.05 compared to the non-irradiated control groups (white bars); #*p*<0.05 compared to the irradiated control groups (black bars).

The possible anti-inflammatory activity of formulation containing NGN in UVB-induced skin inflammation in hairless mice was evaluated. Firstly, it was observed that UVB irradiation induced a significant increase of skin edema in untreated irradiated animals and irradiated animals treated with control formulation (Fig 3). NGN containing formulation did not alter skin edema *per se*. On the other hand, formulation-containing NGN inhibited UVB-induced skin edema (Fig 3).

### NGN containing formulation inhibits UVB irradiation-induced cytokine production

**Fig 4:**
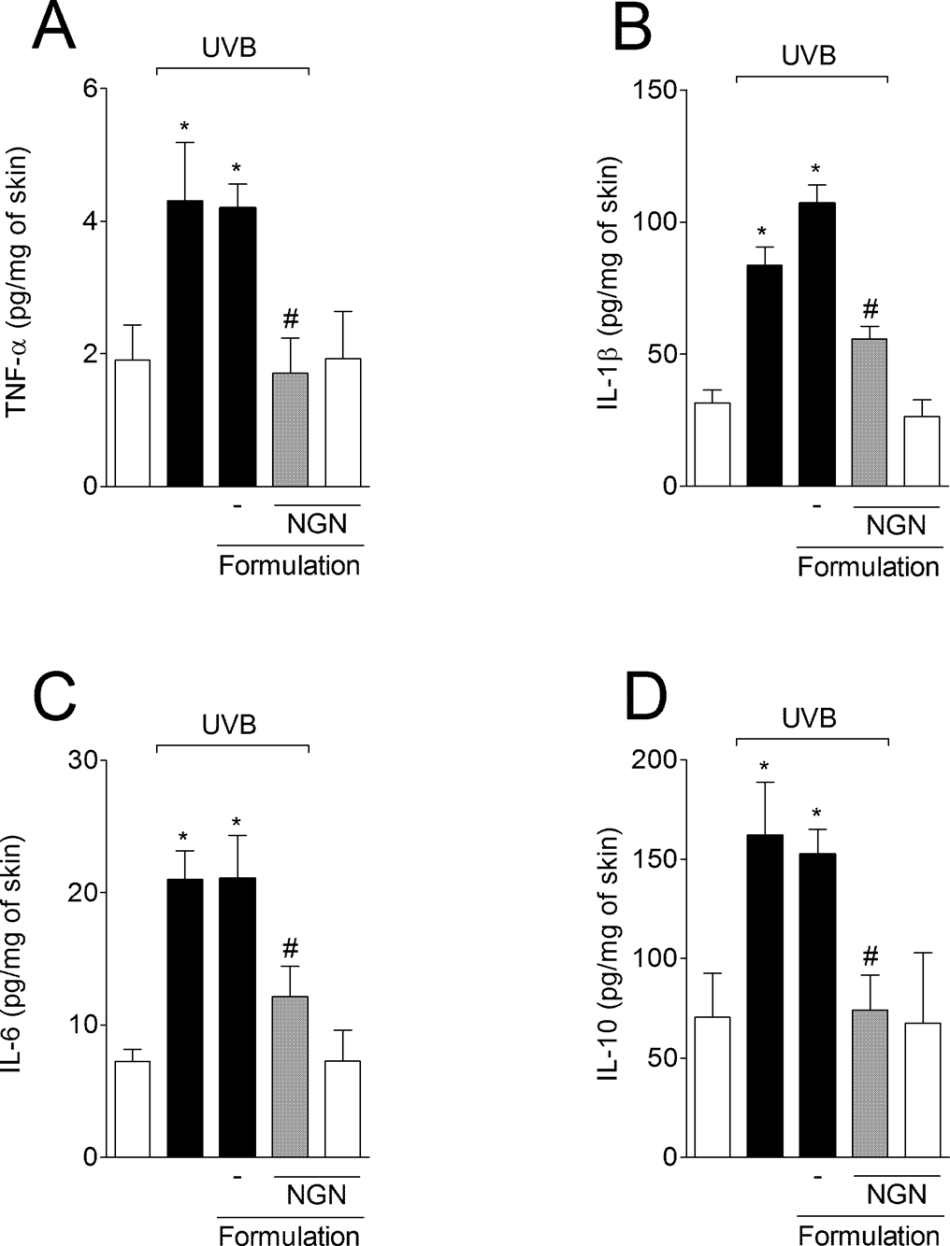
Naringenin (NGN) containing formulation inhibits UVB irradiation-induced cytokine production. The levels of TNF-α (A), IL-1β (B), IL-6 (C) and IL-10 (D) were determined in samples collected 4 h after the end of irradiation. Bars represent means ± SEM of 5 mice per group per experiment and are representative of two separate experiments. *p<0.05 compared to the non-irradiated control groups (white bars); #*p*<0.05 compared to the irradiated control groups (black bars).

UVB irradiation induced significant increase of pro-inflammatory cytokines TNF-α (Fig 4A), IL-1β (Fig 4B) and IL-6 (Fig 4C), and anti-inflammatory cytokine IL-10 (Fig 4D) production compared to non-irradiated mice. Formulation containing NGN inhibited the production of all cytokines evaluated (Fig 4). Formulation without NGN in UVB-irradiated mice and NGN containing formulation in naive mice induced no effect.

### NGN containing formulation prevents UVB irradiation-induced decrease of antioxidant capacity

**Fig 5:**
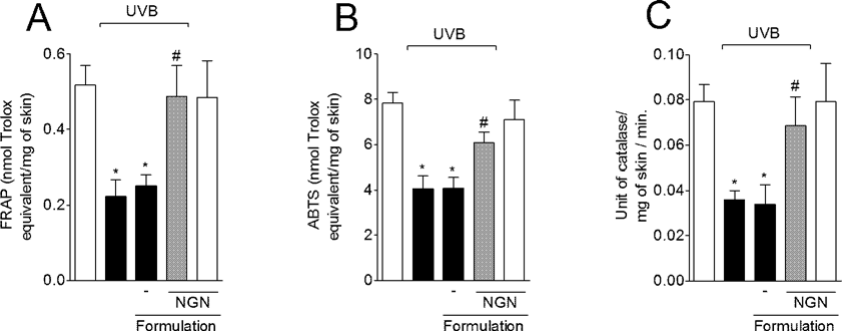
Effect of naringenin (NGN) containing formulation on antioxidant capacity of skin after UVB irradiation. The antioxidant capacity was measured using FRAP (A) and ABTS (B) assays in samples collected 12 h after the end of irradiation. (C) Catalase activity was determined in samples collected 2 h after the end of irradiation. Bars represent means ± SEM of 5 mice per group per experiment and are representative of two separate experiments. *p<0.05 compared to the non-irradiated control groups (white bars); #*p*<0.05 compared to the irradiated control groups (black bars).

UVB irradiation decreased the skin ferric reducing ability (FRAP) (Fig 5A) and ABTS radical scavenging (Fig 5B) activities compared to non-irradiated mice. In turn, the treatment with NGN containing formulation inhibited UVB irradiation-induced depletion of FRAP and ABTS activities, which were maintained at similar levels of the non-irradiated control group (Fig 5A and 5B). UVB irradiation also reduced the skin catalase activity, which was unaffected by control formulation without NGN. In line with the FRAP and ABTS results, NGN containing formulation was able to inhibit catalase activity depletion (Fig 5C). Formulation without NGN in UVB-irradiated mice and NGN containing formulation in naive mice induced no effect.

### NGN containing formulation inhibits UVB irradiation-induced O_2_^•-^ production and LPO of skin

**Fig 6:**
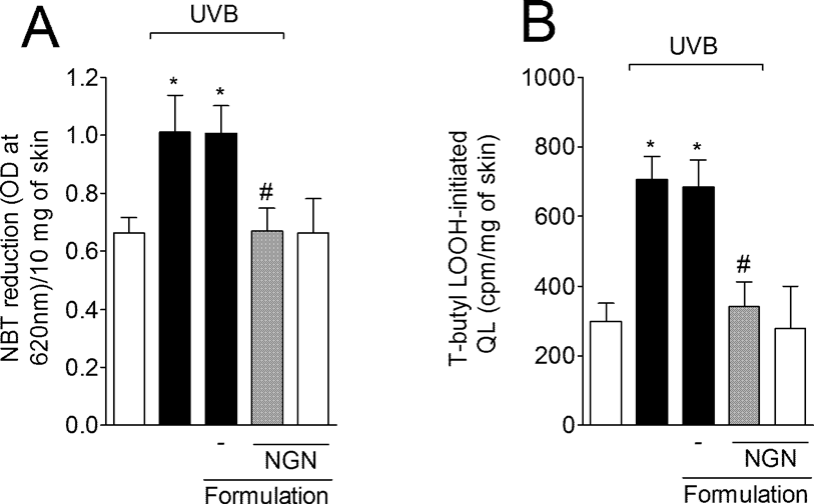
Naringenin (NGN) containing formulation inhibits superoxide anion generation and lipid peroxidation induced by UVB irradiation. (A) Superoxide anion production was measured by nitroblue tetrazolium (NBT) reduction assay in samples collected 2 h after the end of irradiation. (B) Lipid peroxidation was measured by tert-butyl hydroperoxide-(LOOH) initiated chemiluminescence (QL) assay in samples collected 4 h after the end of irradiation. Bars represent means ± SEM of 5 mice per group per experiment and are representative of two separate experiments. *p<0.05 compared to the non-irradiated control groups (white bars); #*p*<0.05 compared to the irradiated control groups (black bars).

UVB irradiation induced O_2_^•-^ production in the skin while treatment with NGN containing formulation inhibited this production reaching non-irradiated control group levels (Fig 6A). UVB irradiation also increased skin LPO while treatment with NGN containing formulation inhibited this parameter reaching similar levels as for non-irradiated control group (Fig 6B). Formulation without NGN in UVB-irradiated mice and NGN containing formulation in naive mice induced no effect.

### NGN containing formulation inhibits UVB irradiation-induced down-regulation of glutathione system components

**Fig 7:**
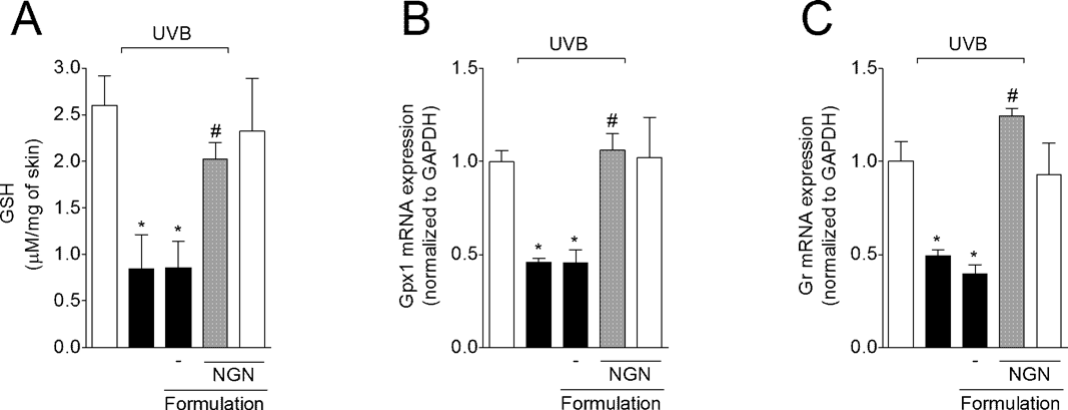
Naringenin (NGN) containing formulation inhibits UVB irradiation-induced down-regulation of glutathione system components. (A) Reduced glutathione (GSH) levels were measured in samples collected 12 h after the end of irradiation. Expression of (B) glutathione peroxidase 1 (Gpx1) and (C) glutathione reductase (Gr) in the skin was measured 4 h after the end of irradiation by qPCR. Bars represent means ± SEM of 5 mice per group per experiment and are representative of two separate experiments. *p<0.05 compared to the non-irradiated control groups (white bars); #*p*<0.05 compared to the irradiated control groups (black bars).

UVB irradiation decreased the GSH levels (Fig 7A), and glutathione peroxidase 1 (Gpx1) (Fig 7B) and glutathione reductase (Gr) (Fig 7C) mRNA expression compared to non-irradiated control group. The topical treatment with NGN containing formulation maintained GSH levels, and Gpx1 and Gr mRNA expression at basal levels (Fig 7A–7C). Formulation without NGN in UVB-irradiated mice and NGN containing formulation in naive mice induced no effect.

### NGN containing formulation inhibits UVB irradiation-induced Nrf2 (nuclear factor erythroid 2-related factor 2) down-regulation and improves heme oxygenase-1 (HO-1) mRNA expression in the skin

**Fig 8:**
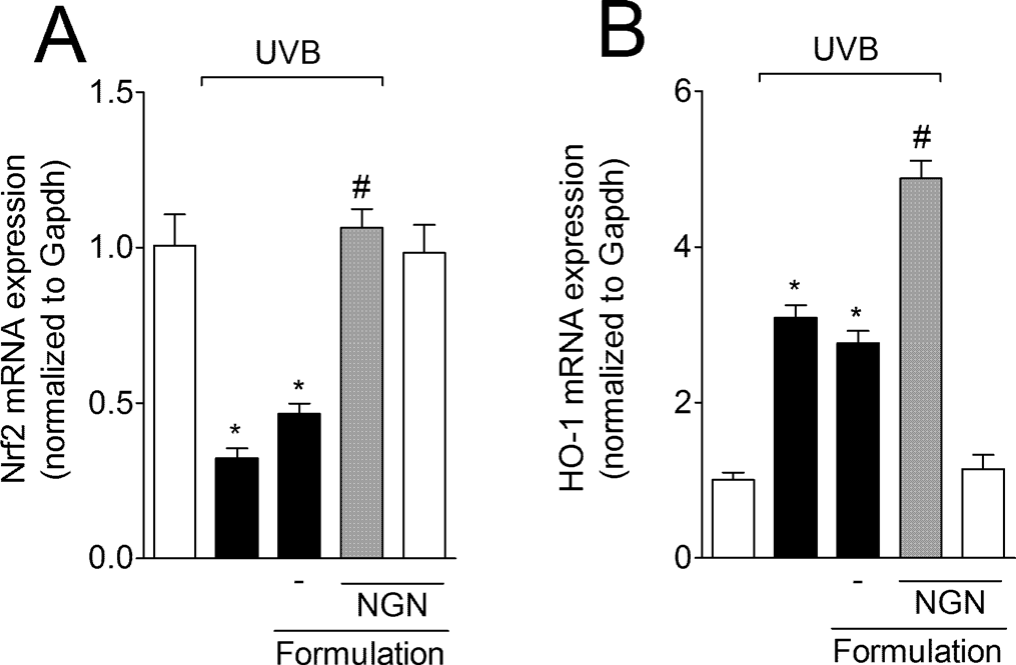
Naringenin (NGN) containing formulation inhibits UVB irradiation-induced Nrf2 down-regulation and improves HO-1 expression in the skin. Expression of (A) Nrf2 and (B) heme oxygenase-1 (HO-1) in the skin was measured 4 h after the end of irradiation by qPCR. Bars represent means ± SEM of 5 mice per group per experiment and are representative of two separate experiments. *p<0.05 compared to the non-irradiated control groups (white bars); #*p*<0.05 compared to the irradiated control groups (black bars).

UVB irradiation reduced the Nrf2 mRNA expression in the skin compared to non-irradiated control group, an effect that was amenable by NGN containing formulation reaching non-irradiated control levels (Fig 8A). Nrf2 induces HO-1 expression, and the enhanced HO-1 activity is a protective response to cellular stress such as inflammation [57]. NGN containing formulation enhanced UVB irradiation-induced HO-1 mRNA expression (Fig 8B). Formulation without NGN in UVB-irradiated mice and NGN containing formulation in naive mice induced no effect.

## Discussion

Naringenin (NGN) is a flavonoid with great therapeutic perspectives as anti-inflammatory and antioxidant [38–40,42,43]. We firstly determined the *in vitro* antioxidant properties of NGN selecting a suitable method for the following functional stability studies of three topical formulations containing NGN resulting in the selection of F3 for *in vivo* testing. F3 containing NGN inhibited UVB-induced skin inflammation and oxidative stress in mice. To our knowledge, this is the first study demonstrating the development and *in vivo* efficacy of a topical formulation containing NGN for the treatment of UVB-induced oxidative stress and inflammation.

Our *in vitro* results on antioxidant properties of NGN show that it inhibits LPO in non-enzymatic systems in part by scavenging cationic (ABTS) and anionic (^•^OH) radicals. Of note, the antioxidant activity of NGN was more prominent in ABTS radical (IC_50_ = 0.71 μg/mL or 2.60 μM) than in ^•^OH radical (IC_50_ = 183 μg/mL or 672 μM) scavenging assays. Thus, suggesting that electron donation accounts for a direct antioxidant mechanism of NGN rather than hydrogen donation. Accordingly, we observed that NGN inhibits iron-independent LPO, which involves initial products of LPO such as hydroperoxide lipids formation [48]. NGN also inhibits iron-dependent LPO, which is mediated by peroxyl and alkoxyl radicals during propagation and termination of LPO. The IC_50_ of iron-independent LPO was 101 μg/mL (370 μM) and of iron-dependent LPO was 159 μg/mL (584 μM). Therefore, NGN can inhibit these three levels of lipid peroxidation in concentrations that are much higher than that necessary to scavenge ABTS radical. In line with these results, NGN scavenges the ABTS radical at an IC_50_ of 7.9 μM, and inhibits peroxyl- and hydroxyl-induced LPO with an IC_50_ of 1210 μM and 230 μM, respectively [58]. The lack of an *orto*-hydroxyl structure in the B ring and conjugation provided by the 2,3-double bond with the 4-oxo group explain the relatively low capacity of NGN in scavenging anionic radicals [59,60]. Therefore, suggesting that the powerful anti-inflammatory activity of NGN reported elsewhere [42,43,61] occurs by mechanisms other than solely scavenging free radicals.

The effectiveness of flavonoids in inhibiting disease depends on the pharmacokinetics of each compound preserving the bioavailability [62]. NGN have weak water solubility and undergo degradation in the acidic stomach environment [63]. These properties cause low dissolution rates from oral dosage forms, resulting in a low absorption and bioavailability. On the other hand, topical formulations prevent gastrointestinal degradation and represent a promising route of administration for these compounds. An important step in the development of novel products is the stability evaluation of active principles of formulations stored at varied climatic conditions for given times. It provides information about the shelf life of pharmaceutical products as well as the conditions for their storage. Therefore, stability testing represents a crucial part of the testing program since the instability of the product modifies essential requisites, i.e., quality, efficacy, and safety [25,56].

A stable emulsion maintains adequate proportions between its components and the interphase surface even after being exposed to tension resulting from factors such as temperature, agitation, and acceleration of gravity [56]. The formulations developed in this study remained physically stable (no phase separation), and the pH values remained satisfactory in two storage conditions evaluated. Measuring the pH is necessary to detect pH alterations during a storage period, ensuring that the pH value is compatible with the components of the formulation and with the place of application, avoiding irritation [25,56]. F1 and F2 formulations stored at 40±2°C/75±5% RH presented decrease of consistency while F3 remained unaltered. F1 lost its antioxidant activity after six months in both storage conditions. Moreover, the antioxidant activity of F2 was lower than NGN raw material since the beginning of the study, indicating possible incompatibilities between F2 and NGN. Of note, F2 contains Hostacerin^®^, which is a complex mixture of excipients increasing the range of possible incompatibilities. F3 was the most stable formulation. Thus, we used F3 for evaluation of *in vivo* efficacy of NGN-containing formulation and demonstrated that this product was effective against skin inflammation and oxidative stress caused by UVB irradiation.

The pathogenesis of skin inflammation depends on the ROS-driven production of cytokines such as TNF-α, IL-1β, and IL-6 [64]. The release of these cytokines plays a central role in inflammation and skin damage after UVB exposure [65,66]. In fact, cytokine inhibition is considered a therapeutic approach to control skin cancer progression and metastasis, and other skin diseases such as psoriasis [64,67–69]. Herein, we demonstrated that the NGN containing formulation inhibited skin edema after UVB irradiation as well as the production of inflammatory cytokines (TNF-α, IL-1β, and IL-6). NGN containing formulation also inhibited UVB-induced increases of IL-10 production in the skin. IL-10 is an anti-inflammatory cytokine in models of acute inflammation [33,70]. On the other hand, IL-10 has been associated with UVB-induced immunosuppression and DNA damage and diminishing its production prevents these events [71,72]. IL-10 is co-produced with pro-inflammatory cytokines to limit the inflammatory response [73]. In this sense, the inhibition of TNF-α, IL-1β, and IL-6 production might have as a consequence the reduction of IL-10 levels.

The NGN containing formulation inhibited, for instance, the UVB-induced catalase activity impairment and production of lipid peroxidation products. Taking into account this antioxidant effect, we aimed to describe, at least in part, the effects of the NGN containing formulation in UVB irradiation-induced production of ROS. O_2_^•-^ production represents an important step for the beginning of oxidative stress during inflammation [8]. O_2_^•-^ is essential to induce cytokine production [17,74]. The NGN containing formulation inhibited UVB irradiation-induced production of O_2_^•-^ and inflammatory cytokines. *In vitro*, NGN exhibits low O_2_^•-^ radical scavenging ability, reaching 12% inhibition at 1 mM [75]. In this study, the authors used a non-enzymatic source of O_2_^•-^ (phenazine methosulfate/NADH) and NBT reduction test to evaluate O_2_^•-^ scavenging ability [75]. Therefore, this evidence [63] suggests that our observations regarding the inhibition of NBT reduction *in vivo* depend on the anti-inflammatory activity of NGN rather than to an O_2_^•-^ scavenging activity. Possible NGN mechanisms to inhibit superoxide anion production include: 1) inhibition of NADPH oxidase activity [76]. In fact, mice lacking gp91phox expression do not present UVB-induced skin inflammation, gp91phox is the subunit of NADPH oxidase that catalyzes O_2_ reduction and represents an important source of O_2_^•-^ during the inflammatory response [65]. 2) NGN inhibition of O_2_^•-^ generation was 18-fold greater in an enzymatic xanthine oxidase system (IC_50_ = 4.4 μM) compared to the non-enzymatic phenazine methosulfate/NADH reaction system (IC_50_ = 94.7 μM) [58]. Thus, suggesting that NGN can modulate the production and activity of enzymes related to oxidative stress. 3) NGN inhibits superoxide dismutase depletion induced by a wide range of pro-oxidant stimuli and in several organs, including kidney [38], liver [77], and brain [78]. Therefore, superoxide dismutase production may represent an additional mechanism that contributes to reducing O_2_^•-^ production in mice treated with NGN containing formulation. 4) UVB-induced inflammation-related enzymes such as cyclooxygenase and lipoxygenase also produce O_2_^•-^ in the presence of NADH and NADPH [13]. NGN inhibits LPS-induced macrophage cyclooxygenase-2 expression [79].

It is important to highlight that electrons from the mitochondrial complex I and III of the electron transport chain can leak and react with molecular O_2_ producing O_2_^•-^ upon UVB irradiation [12]. Ionizing radiation induces mitochondrial DNA damage leading to impaired oxidative phosphorylation process and continuous O_2_^•-^ production [80]. This process is likely to occur during UVB irradiation although it lacks actual demonstration in UVB model. Therefore, UVB-induced O_2_^•-^ production occurs from varied sources including NADPH oxidase, cyclooxygenase, lipoxygenase and mitochondria [12,13,65,80].

Several ROS-eliminating systems are present in mammalian tissues to protect cells from ROS overproduction as occurs after UVB irradiation exposition. However, excessive UVB irradiation exposure depletes endogenous antioxidants in the skin, including catalase and Gpx, which are the most important antioxidant enzymes during cell detoxification [5,6,15]. Excessive ROS production after UVB irradiation induces the consumption of GSH, which is the most abundant non-enzymatic antioxidant in the cells [52]. GSH depletion occurs directly by ROS production, but it can also be depleted indirectly because of it is a substrate for Gpx during detoxification [81]. NGN-containing formulation inhibited UVB-induced GSH consumption, but also inhibited the depletion of Gpx1 mRNA expression in the skin, which could be seam as unexpected and controversial results. Nevertheless, NGN-containing formulation also maintained Gr mRNA expression at basal levels. These results suggest that NGN containing formulation (F3) keeps the basal levels of GSH by maintaining a balanced production of Gpx1 (oxidizes GSH to remove ROS-related products) and Gr (recycles GSH). Therefore, topical NGN treatment is a successful strategy to protect the skin from oxidative damage induced by UVB irradiation. In line with our results, the antioxidant activity of NGN was related to the inhibition of GSH, Gpx1 and Gr depletion in other studies [38,41,43,78].

The transcription factor Nrf2 is the major redox-dependent regulator of the expression of detoxifying and antioxidant enzymes. Inhibition of Nrf2 activity potentiates UVB irradiation-induced skin damage due to enhanced production of inflammatory mediators, including IL-1β, IL-6 [82] and metalloproteinase-9 [83]. NGN containing formulation inhibited UVB-induced reduction of Nrf2 mRNA expression, suggesting that NGN enables the skin to respond more efficiently to oxidative stress induced by UVB in a Nrf2-dependent manner. Corroborating this, treatment with NGN-containing formulation enhanced UVB-induced HO-1 mRNA expression. In fact, Nrf2 induces HO-1 expression in the skin in response to oxidative stress. HO-1 is a stress-responsive antioxidant enzyme essential to maintaining cellular resistance during stress conditions, which may explain why HO-1 production increases while other antioxidant enzymes are inhibited [57]. Moreover, enhancing HO-1 expression is a promising therapeutic approach to inhibit skin damage after irradiation exposure [84].

The present study aimed to develop a topical formulation containing NGN active *in vivo* against UVB irradiation. Nevertheless, it is important to mention that additional mechanisms of action of NGN containing formulation could be determined. For instance, in other disease context NGN acts by inhibiting/ suppressing the activation of the MAPKs (mitogen-activated protein kinases) p38 (p38 mitogen-activated protein kinase), JNK (c-Jun N-terminal kinase) and ERK (extracellular-signal-regulated kinase), and transcription factors STAT (signal transducer and activator of transcription), NF-kB (nuclear factor kappa-light-chain-enhancer of activated B cells) and AP-1 (activating protein-1) [85–87], and reduction the NADPH oxidase activity [76]. All these NGN targets have been demonstrated to be important in the pathophysiology of UVB irradiation and might contribute to the efficacy of NGN containing formulation developed in this study. Additionally, it remains to be determined if the local skin antioxidant and anti-inflammatory effects of topical formulation containing NGN modulates the systemic effects of UVB irradiation such as the activation of the hypothalamic–pituitary–adrenal axis [2,3].

## Conclusions

Topical formulations containing NGN were prepared and analyzed regarding their physicochemical characteristics and antioxidant activity in varied storage conditions during six months. F3 stability compared to the other formulations rendered it suitable for further studies. Topical application of F3 in the dorsal skin of hairless mice protected from UVB irradiation-induced skin damage. In fact, F3 containing NGN maintained the mRNA expression of cellular antioxidant components (Gpx1, Gr, Nrf2) and induced HO-1 mRNA expression. These molecular effects accounted for the improvement of antioxidant capacity in the skin observed as ferric reducing ability, ABTS radical scavenging capacity, the levels of reduced glutathione and catalase activity and inhibition of O_2_^•-^ radical production. F3 containing NGN also inhibited UVB irradiation-induced lipid hydroperoxides, edema and cytokine production (TNF-α, IL-1β, IL-6, and IL-10). Our results suggest that topical treatment with NGN containing formulation is a potential product for the treatment of UVB-related skin diseases to explore. The present data open the possibility for future studies to addressing all modes of action of the topical formulation containing NGN as well as novel applications of this formulation for other inflammatory and oxidative skin diseases.
